# Purslane Weed (*Portulaca oleracea*): A Prospective Plant Source of Nutrition, Omega-3 Fatty Acid, and Antioxidant Attributes

**DOI:** 10.1155/2014/951019

**Published:** 2014-02-10

**Authors:** Md. Kamal Uddin, Abdul Shukor Juraimi, Md Sabir Hossain, Most. Altaf Un Nahar, Md. Eaqub Ali, M. M. Rahman

**Affiliations:** ^1^Institute of Tropical Agriculture, University Putra Malaysia, Serdang, Malaysia; ^2^Bangladesh Agricultural University, Mymensingh 2202, Bangladesh; ^3^Malaysia Nanotechnology and Catalysis Research Center, University of Malaya, 50603 Kuala Lumpur, Malaysia; ^4^Bangabadhu Sheikh Mujibur Rahman Agricultural University, Gazipur 1706, Bangladesh

## Abstract

Purslane (*Portulaca oleracea* L.) is an important plant naturally found as a weed in field crops and lawns. Purslane is widely distributed around the globe and is popular as a potherb in many areas of Europe, Asia, and the Mediterranean region. This plant possesses mucilaginous substances which are of medicinal importance. It is a rich source of potassium (494 mg/100 g) followed by magnesium (68 mg/100 g) and calcium (65 mg/100 g) and possesses the potential to be used as vegetable source of omega-3 fatty acid. It is very good source of alpha-linolenic acid (ALA) and gamma-linolenic acid (LNA, 18 : 3 w3) (4 mg/g fresh weight) of any green leafy vegetable. It contained the highest amount (22.2 mg and 130 mg per 100 g of fresh and dry weight, resp.) of alpha-tocopherol and ascorbic acid (26.6 mg and 506 mg per 100 g of fresh and dry weight, resp.). The oxalate content of purslane leaves was reported as 671–869 mg/100 g fresh weight. The antioxidant content and nutritional value of purslane are important for human consumption. It revealed tremendous nutritional potential and has indicated the potential use of this herb for the future.

## 1. Introduction

Purslane (*Portulaca oleracea *L.) deserves special attention from agriculturalists as well as nutritionists. Purslane is a common weed in turfgrass areas as well as in field crops [1, 2]. Many varieties of purslane under many names grow in a wide range of climates and regions. Purslane has wide acceptability as a potherb in Central Europe, Asia, and the Mediterranean region. It is an important component of green salad and its soft stem and leaves are used raw, alone, or with other greens. Purslane is also used for cooking or used as a pickle. Its medicinal value is evident from its use for treatment of burns, headache, and diseases related to the intestine, liver, stomach, cough, shortness of breath, and arthritis. Its use as a purgative, cardiac tonic, emollient, muscle relaxant, and anti-inflammatory and diuretic treatment makes it important in herbal medicine. Purslane has also been used in the treatment of osteoporosis and psoriasis.

Recent research demonstrates that purslane has better nutritional quality than the major cultivated vegetables, with higher beta-carotene, ascorbic acid, and alpha-linolenic acid [3]. Additionally, purslane has been described as a power food because of its high nutritive and antioxidant properties [4]. Different varieties, harvesting times, and environmental conditions can contribute to purslane's nutritional composition and benefits [5].

Purslane is popular as a traditional medicine in China for the treatment of hypotension and diabetes. Scientifically, it is not proven to have antidiabetic effects, but still people use it for this purpose. An experiment has been carried out for the extraction of crude polysaccharide(s) from purslane to investigate the hypoglycemic effects of these constituents with animal tests for the use of this plant in the treatment of diabetes [6].

Purslane is a very good source of alpha-linolenic acid. Alpha-linolenic is an omega-3 fatty acid which plays an important role in human growth and development and in preventing diseases. Purslane has been shown to contain five times higher omega-3 fatty acids than spinach. Omega-3 fatty acids belong to a group of polyunsaturated fatty acids essential for human growth, development, prevention of numerous cardiovascular diseases, and maintenance of a healthy immune system [7]. Our bodies do not synthesise omega-3 fatty acids. Therefore omega-3 fatty acids must be consumed from a dietary source. Omega-3 fatty acids contain 18 to 24 carbon atoms and have three or more double bonds within its fatty acid chain [8]. Fish is the richest source of omega-3 fatty acids. Health authorities highly recommend that we consume fish regularly to meet our bodies' requirements of omega-3 fatty acids, as other sources are limited and do not supply nearly as much omega-3 fatty acids [9]. Purslane has recently been identified as the richest vegetable source of alpha-linolenic acid, an essential omega-3 fatty acid [10]. The lack of dietary sources of omega-3 fatty acids has resulted in a growing level of interest to introduce purslane as a new cultivated vegetable [11, 12]. Purslane flourishes in numerous biogeographical locations worldwide and is highly adaptable to many adverse conditions such as drought, saline, and nutrient deficient conditions [13].

*Distribution.* It is reported that purslane was a common vegetable of the Roman Empire. Origin of purslane is not certain, but existence of this plant is reported about 4,000 years ago. The succulent stems and fleshy leaves of purslane reflect that it may have originated and adapted to desert climates of the Middle East and India. It can be found in Europe, Africa, North America, Australia, and Asia [14]. 

*Botanical Classification. Portulaca oleracea* is s cosmopolitan species and the genus *Portulaca *belongs to the family Portulacaceae, a small family with 21 genera and 580 species, and is cosmopolitan in distribution, occurring especially in America with some species found in Arabia [15]. Purslane plants are succulent, annual herbaceous, and erect or decumbent up to 30 cm high. Purslane is botanically known as *Portulaca oleracea* and is also called portulaca. 

*Habitat.* It grows well in orchards, vineyards, crop fields, landscaped areas, gardens, roadsides, and other disturbed sites. 

*Stem.* Stems are cylindrical, up to 30 cm long, 2-3 mm in diameter, green or red, swollen at the nodes, smooth, glabrous apart from the leaf axils, and diffusely branched, and the internodes are 1.5–3.5 cm in length. 

*Leaf.* Purslane leaves are alternate or subopposite, flat, fleshy, having variable shapes, obovate, 1–5 cm long, 0.5–2 cm across, obtuse or slightly notched at the apex, tapering at base, sessile or indistinctly petiolate, glabrous, smooth, and waxy on the upper surface, with entire margin, small stipules, and cluster of hairs up to 1 mm long. Leaves are egg to spatula shaped, succulent, and stalkless or have very short stalks, about 5–30 mm long, and sometimes their edges are red-tinged. Leaves are green or green with red margin. 

*Seedling.* Cotyledons (seed leaves) are egg shaped to oblong, hairless, succulent, about 2–5 mm long, and sometimes tinged red. 

*Flower.* Flowering initiates during May to September. Flowers originate as single or clusters of two to five at the tips of stems. The flowers are minute or small having orange yellow, purple, or white pink color with five petals and typically open only on hot and sunny days from mid-morning to early afternoon. 

*Fruit.* Fruit consists of almost round to egg-shaped capsules, usually about 4–8 mm long that open around the middle to release the seeds. Seeds are tiny, less than 1 mm in diameter, circular to egg shaped, flattened, and brown to black with a white point of attachment. Numerous seeds are produced.

## 2. Health Benefits of Purslane

### 2.1. Nutrition

It is rich in vitamin A which is a natural antioxidant value. It can play role in vision healthy mucus membranes and to protect from lung and oral cavity cancer. Purslane contains the highest content of vitamin A among green leafy vegetables. It also contains vitamin C and B-complex vitamins like riboflavin, niacin, and pyridoxine. It provides highest dietary minerals such as potassium (494 mg/100 g) followed by magnesium (68 mg/100 g), calcium (65 mg/100 g), phosphorus (44 mg/100 g), and iron (1.99 mg/100 g) (Table 1).

The range of Ca, Mg, K, Fe, and Zn from the young stage to mature plants was from 1612 ± 27 to 1945 ± 30 mmol kg^−1^ DW, 2127 ± 23 to 2443 ± 27 mmol kg^−1^ DW, 1257 ± 10 to 1526 ± 31 mmol kg^−1^ DW, 218 ± 8 to 262 ± 3 mmol kg^−1^ DW, and 128 ± 2 to 160 ± 1 mmol kg^−1^ DW, respectively. On the other hand, the Na and Cl concentrations in leaves were higher at the young stage and lower at the mature stage. The Na and Cl concentrations ranged from 356 ± 4 to 278 ± 8 mmol kg^−1^ DW and from 82 ± 2 to 53 ± 2 mmol kg^−1^ DW, respectively [16].

### 2.2. Omega-3 Fatty Acid

Purslane is one of the richest green plant sources of omega-3 fatty acids. It has lower the cholesterol and triglyceride levels, raise the beneficial high density lipoprotein. Moreover, the ability of omega-3 fatty acids to decrease the thickness of the blood may be advantageous in the treatment of vascular diseases [3]. Unlike fish oils with their high cholesterol and calorie content, purslane also provides an excellent source of the beneficial omega-3 fatty acids without the cholesterol of fish oils, since it contains no cholesterol. There are 3 varieties of purslane, namely, the green, golden, and a large-leaved golden variety [17, 18]. Important sources of omega-3 fatty acids are summarized in Table 2. It has a low incidence of cancer and heart disease, possibly due in part to purslane's naturally occurring omega-3 fatty acids [19].

Purslane is best used for human consumption as a green vegetable rich in minerals and omega-3 fatty acids [20]. Omega-3 fatty acid is a precursor of a specific group of hormones. It may offer protection against cardiovascular disease, cancers, and a number of chronic diseases and conditions throughout the human life. The antioxidant enzymes such as GPx, GR, SOD, and GST take part in maintaining glutathione homeostasis in tissues. Also, increased levels of GPx, GR, GST, CAT, and SOD were found to correlate with elevated glutathione level and depressed MDA and NO in rats, thus showing the antioxidant activity of purslane.

Purslane leaves contain higher contents of alpha-linolenic acid (18 : 3 w3), alpha-tocopherol, ascorbic acid and glutathione than the leaves of spinach. It grows in growth chambers containing seven times higher contents of alpha-tocopherol than that found in spinach. One hundred grams of fresh purslane leaves (one serving) contains about 300–400 mg of 18 : 3 w3; 12.2 mg of alpha-tocopherol, 26.6 mg of ascorbic acid, 1.9 mg of beta-carotene, and 14.8 mg of glutathione [21].

Purslane has the highest level of alpha-linolenic which is an omega 3 fatty acid essential for human nutrition compared to any leafy green vegetable. A 100 g sample of purslane contains 300–400 mg of alpha-linolenic acid (ALA). It also has 0.01 mg per gram of eicopentanoic acid (EPA), which is not present at all in flax oil. This would provide 1 mg of EPA for a 100 g portion of purslane or 10 mg for a kg (2.2 pounds), or 1 g for 100 kg (220 pounds) of sample.

Purslane is the richest source of gamma-linolenic acid (LNA, 18 : 3 w3) (4 mg/g fresh weight) of any green leafy vegetable. It also contains a small amount of eicosapentaenoic acid (EPA. 20 : 5 w3) (0.01 mg/g fresh weight) [21]. Subsequently, purslane contained 18 : 3 w3 20 : 5 w3 and 22  :  6 w3 (docosahexaenoic acid, DHA) as well as 22 : 5 w3 (docosapentaenoic acid, DPA) [22].

Selected food sources of omega-3 fatty acids are illustrated in Table 2. Rapeseed oil, walnut oil, butternuts, and wheat germ oil are excellent sources (6.8–11.1 g/100 g fresh weight) of omega-3 fatty acids. Good sources (0.6–3.2 g/100 g fresh weight) of these fats include green soybean, soybean kernels, beechnut, and oat germ. Cabbage, cauliflower, broccoli, strawberries, spinach, garden pea, corn, and common dry bean are additional sources of omega-3 fatty acids. These foods contain a smaller (0.1–0.3 g/100 g fresh weight) level of omega-3 fatty acids.

Purslane leaves contained higher amounts of alpha-linolenic (18 : 3 w3) than stem fractions, whereas 20 : 5w3 was higher in stem fractions [22] (Table 3).

Leaves of purslane grown both in the controlled growth chamber and in the wild contained higher amount of alpha-linolenic fatty acid (18 : 3 w3) than that of spinach leaves. The highest amount (3.41 mg/g) of alpha-linolenic acid was recorded in growth chamber grown purslane, which was seven times higher than that of spinach leaves (0.48 mg/g) (Table 4).

### 2.3. Lipid Content and Fatty Acid Composition

All fractions contained very low lipid content with 0.47% in stems, 0.51% in leaves, and 0.54% in the flowers (Table 5). In general, polyunsaturated fatty acids (PUFAs) were found to be most abundant in all fractions, followed by saturated (SFAs) and monounsaturated fatty acids (MUFAs). The most predominant fatty acids were 18:3n-3 (50%) in the leaf, 18:3n-6 (46%) in the stem, and 18:2n-6 (30% of total fatty acid) in the flowers. ALA content ranged from 149 to 523 mg (100 g sample) in stems and leaves, respectively. An interesting finding in this study was that 18:3n-6 was found at high levels in all fractions, accounting for 46% in stems, 13% in leaves, and 10% in flowers [23].

### 2.4. Antioxidants

The TPC in cultivars of *P. oleracea* ranged from 127 ± 13 to 478 ± 45 mg GAE/100 g fresh weight of plant. The IC50 ranged from 0.89 ± 0.07 to 3.41 ± 0.41 mg/mL, the AEAC values ranged from 110 ± 14 to 430 ± 32 mg AA/100 g, and the FRAP values ranged from 0.93 ± 0.22 to 5.10 ± 0.56 mg GAE/g [24] (Lim and Quah 2007). DPPH scavenging (IC50) capacity ranged from 1.30 ± 0.04 to 1.71 ± 0.04 mg/mL, while the ascorbic acid equivalent antioxidant activity (AEAC) values were from 229.5 ± 7.9 to 319.3 ± 8.7 mg AA/100 g, the total phenol content (TPC) varied from 174.5 ± 8.5 to 348.5 ± 7.9 mg GAE/100 g, AAC varied from 60.5 ± 2.1 to 86.5 ± 3.9 mg/100 g, and FRAP ranged from 1.8 ± 0.1 to 4.3 ± 0.1 mg GAE/g [16].

Higher amounts of alpha-tocopherol, ascorbic acid, and beta-carotene were observed in the leaves of purslane grown both in the growth chamber and in the wild, compared to the composition of spinach leaves (Table 6). Growth chamber grown purslane contained the highest amount (22.2 mg and 130 mg per 100 g of fresh and dry weight, resp.) of alpha-tocopherol and ascorbic acid (26.6 mg and 506 mg per 100 g of fresh and dry weight, resp.), whereas beta-carotene was slightly higher in spinach.

Vitamin C (ascorbic acid) and beta-carotene have been reported to possess antioxidant activity, because of their ability to neutralize free radicals, and have the potential to prevent cardiovascular disease and cancer [25]. Leaves had the highest content of beta-carotene, ascorbic acid, and DPPH, followed by flowers and stems (Table 7). Thai wild purslane contained almost 10 times higher beta-carotene and ascorbic acid [3, 4, 26] content than other varieties. The beta-carotene content in the leaf was two times higher than in the stems and slightly higher than in the flowers. This finding is in agreement with the data on Australian purslane, where the beta-carotene content in the leaf was higher than in the stem [3]. Purslane is amongst the group of plants with high oxalate contents. Melatonin is a ubiquitous and versatile molecule that exhibits most of the desirable characteristics of a good antioxidant [27]. The oxalate content of purslane leaves was reported as 671–869 mg/100 g fresh weight [28, 29].

## 3. Conclusion

As a significant source of omega-3 oils, *P. oleracea *could yield considerable health benefits to vegetarian and other diets where the consumption of fish oils is excluded. Scientific analysis of its chemical components has shown that this common weed has uncommon nutritional value, making it one of the potentially important foods for the future. Presence of high content of antioxidants (vitamins A and C, alpha-tocopherol, beta-carotene, and glutathione) and omega-3 fatty acids and its wound healing and antimicrobial effects as well as its traditional use in the topical treatment of inflammatory conditions suggest that purslane is a highly likely candidate as a useful cosmetic ingredient.

## Figures and Tables

**Table 1:** Purslane (*Portulaca oleracea*) (Nutritive value per 100 g).

Principle	Nutrient value	Percentage of RDA
Energy	16 Kcal	1.5%
Carbohydrates	3.4 g	3%
Protein	1.30 g	2%
Total Fat	0.1 g	0.5%
Cholesterol	0 mg	0%
Vitamins		
Folates	12 *μ*g	3%
Niacin	0.480 mg	3%
Pantothenic acid	0.036 mg	1%
Pyridoxine	0.073 mg	5.5%
Riboflavin	0.112 mg	8.5%
Thiamin	0.047 mg	4%
Vitamin A	1320 IU	44%
Vitamin C	21 mg	35%
Electrolytes		
Sodium	45 mg	3%
Potassium	494 mg	10.5%
Minerals		
Calcium	65 mg	6.5%
Copper	0.113 mg	12.5%
Iron	1.99 mg	25%
Magnesium	68 mg	17%
Manganese	0.303 mg	13%
Phosphorus	44 mg	6%
Selenium	0.9 *μ*g	2%
Zinc	0.17 mg	1.5%

Source: USDA National Nutrient data.

**Table 2:** Plant sources of omega-3 fatty acids (g/100 g).

Category	Fruits/vegetables	Amount (g)
Low	Avocados, California raw	0.1
	Broccoli	0.1
	Strawberries	0.1
	Cauliflower, raw	0.1
	Kale, raw	0.2
	Spinach, raw	0.1
	Peas, garden dry	0.2
	Cowpeas, dry	0.3
	Beans, navy, sprouted, cooked	0.3
	Corn, germ	0.3

Medium	Bean, common dry	0.6
	Leeks, freeze-dried, raw	0.7
	Wheat, germ	0.7
	Spirulina, dried	0.8
	Purslane	0.9
	Oat, germ	1.4
	Beachnuts	1.7
	Soybeans kernels, roasted	1.5
	Soybeans, green	3.2

High	Soybean oil	6.8
	Walnuts, Persian, English	6.8
	Wheat germ oil	6.8
	Butternuts	8.7
	Walnut oil	10.4
	Rapeseed oil (New Puritan Oil)	11.1

Source: Bulletin, US Department of Agriculture Provisional table on the content of omega-3 fatty acids and other fat components in selected foods (HNIS/PT-103).

**Table 3:** Composition of selected fatty acids in purslane (*Portulaca oleracea*) (% of total FA)^a^.

Omara-Alwala et al., 1991 [22]				Simopoulos and Salem, 1986 [10]
Fatty acid	Leaf	Stem	Whole plant	Whole plant
18.3-omega-3	41.4–66.4	2.4–5.9	28.4–42.5	47.6
20.5-omega-3	0.8–12.6	18.6–35.5	6.4–21.5	0.1
22.3-omega-3	1.4–3.3	trace	1.0–3.0	—
22.6-omega-3	0.3–6.4	trace	0.6–5.6	—

^a^Results from Omara-Alwala et al., 1991 [22], and Simopoulos and Salem, 1986 [10], expressed as mg of FA per kg or g of net weight.

**Table 4:** Fatty acid profiles in total lipid extracts from leaves of purslane and spinach.

Fatty acid	Chamber grown purslane		Wild purslane		Spinach	
	Dry wt%	mg/g fresh wt	Dry wt%	mg/g fresh wt	Dry wt%	mg/g fresh wt
18.0	1.12	0.064	0.95	0.048	0.78	0.007
18.1	4.99	0.016	2.13	0.10	2.04	0.018
18.2	16.99	0.968	13.45	0.70	11.70	0.10
18.3	59.87	3.41	63.78	3.22	53.85	0.48

Source: Simopoulos et al., 1992 [21].

**Table 5:** Fatty acid composition of purslane fractions.

Fatty acid	Composition (% of total fatty acids)		
	Leaf	Stem	Flower
15:0	0.39	—	1.01
16:0	13.09	16.90	19.30
18:0	2.29	7.75	4.51
Total SFA	**16.42**	**24.64**	**24.52**
16:1	0.54	0.51	0.90
18:1	4.29	3.38	12.30
Total MUFA	**4.83**	**3.89**	**13.20**
18:2n-6	14.46	9.70	30.11
18:3n-6	13.25	45.57	9.68
18:3n-3	49.70	15.62	21.01
20:0	0.21	0.11	0.29
22:0	0.19	0.16	0.10
24:0	1.12	0.31	1.09
Total PUFA	**78.75**	**71.47**	**62.28**
Lipid content (%)	0.51	0.47	0.54

Source: Siriamornpun and Suttajit, 2010 [23].

**Table 6:** Antioxidant content of purslane and spinach leaves.

Name	Alpha-tocopherol		Ascorbic acid		Beta-carotene	
	mg/100 g (fresh wt.)′	mg/100 g (dry wt.)	mg/100 g (fresh wt.)′	mg/100 g (dry wt.)	mg/100 (g fresh wt.)′	mg/100 g (dry wt.)
Chamber grown purslane	22.2	230	26.6	506	1.9	38.2
Wild purslane	8.2	170	23.0	451	2.2	43.5
Spinach	1.8	36	21.7	430	3.3	63.5

Source: Simopoulos et al., 1992 [21].

**Table 7:** Ascorbic acid and beta-carotene content and 1,1-diphenyl-2-picrylhydrazyl (DPPH) radical-scavenging activity of different parts of Thai purslane.

Plant parts	Antioxidant compound content		
	Beta-carotene (mg g^−1^ sample)	Ascorbic acid (mg g^−1^ sample)	DPPH (%)
Leaf	0.58	3.99	76.71
Stem	0.29	2.27	90.11
Flower	0.55	2.32	91.01
